# Molecular T2 asthma phenotypes are stable but heterogeneous: the usefulness of periostin for endotyping

**DOI:** 10.3389/falgy.2023.1205115

**Published:** 2023-09-08

**Authors:** Irina Bobolea, Daniela Guillén-Vera, Natividad De las Cuevas-Moreno, Diego Blanco García-Granero, David Loli-Ausejo, Carlos Melero-Moreno

**Affiliations:** ^1^ Department of Allergy, Hospital Clinic Barcelona-Institute for Health Research (IdiBAPS), Barcelona, Spain; ^2^ Department of Allergy, Hospital Universitario 12 de Octubre-Institute for Health Research (i + 12), Madrid, Spain; ^3^ Department of Pulmonology, Hospital 12 de Octubre Institute for Health Research (i + 12), Madrid, Spain

**Keywords:** asthma, endotypes, phenotypes, molecular phenotype, periostin

## Abstract

**Background:**

The stability of molecular T2/non-T2 phenotypes remains uncertain. The objectives of this study were to assess the stability of these phenotypes and the correlation between serum periostin and asthma T2 phenotypes and endotypes.

**Methods:**

Demographics, clinical data, and blood samples were collected. Patients diagnosed with moderate-to-severe asthma were classified into T2 or non-T2 according to previously defined thresholds of blood eosinophilia and serum total IgE levels. Asthma endotype was also determined. After at least 1 year of follow-up, the stability of T2 phenotypes and endotypes was assessed.

**Results:**

A total of 53 patients (72% women), mean age 47 years (range 16–77), were included. In the initial and second evaluations, the T2 phenotype was found in 41.5% and 43.4% of patients and the non-T2 phenotype was found in 58.4% and 56.7%, respectively. The mean [standard deviation (SD), range] serum periostin level was 52.7 (26.2, 22.6–129.7) ng/mL in patients with T2 phenotype, and 39.3 (25.6, 7.7–104.) ng/mL in non-T2 patients (*P* = 0.063). Periostin levels correlated to endotypes (*P* = 0.001): 45.7 (27.9) ng/mL in allergic asthma (*n* = 16 patients), 64.7 (24.9) in aspirin-exacerbated respiratory disease (*n* = 14), 59.0 (27.6) ng/mL in late-onset eosinophilic asthma (*n* = 4), and 28.3 (13.3) ng/mL in non-eosinophilic asthma (*n* = 18).

**Conclusions:**

T2 and non-T2 asthma phenotypes assessed by accessible methods in daily practice are stable over time yet widely heterogeneous. Serum periostin does not discriminate between T2 and non-T2 phenotypes. Nevertheless, its correlation to asthma endotypes may contribute to guide therapies targeting T2 cytokines in a more personalized approach.

## 1. Introduction

Asthma is currently understood as a complex and heterogeneous inflammatory disease comprising several phenotypes and endotypes. It is mainly associated with type 2 (T2) polarization, which produces interleukin-4 (IL-4), IL-5, and IL-13. The development of new biological therapies targeting T2-driven inflammation has aroused great interest and efforts to identify simple, non-invasive biomarkers capable of defining asthma phenotypes in individual patients (1). The current biomarkers of asthma mediated by the so-called T2 pathway [Th2 and ILC2 (group 2 innate lymphoid cells)] (2–4) include eosinophils in sputum and peripheral blood, the exhaled fraction of nitric oxide (FeNO), serum total IgE, specific IgE/skin prick tests to common aeroallergens, and more recently periostin. Current data supporting the use of these biomarkers to stratify patients in clinical practice is incomplete, and the existence of different overlapping phenotypes that influence the response to treatment is disturbing for the treating physician. Molecular phenotyping of asthma based on type 2 inflammation was defined originally by Woodruff et al. (5): using microarray and polymerase chain reaction analyses of airway epithelial brushings, two distinct molecular phenotypes, Th2-high (T2) and Th2-low (non-T2), were identified (6). These subgroups differed significantly in the expression of IL-5 and IL-13 in bronchial biopsies, airway hyperresponsiveness, serum IgE, blood and airway eosinophilia, subepithelial fibrosis, and airway mucin gene expression (6). The T2 phenotype was defined as an IgE level greater than 100 IU/mL and more than 0.14 × 10^9^ eosinophils per liter in the peripheral blood (6, 7). Moreover, the T2 phenotype was later associated with increased circulating periostin, a matricellular protein induced by IL-13 and expressed by airway structural cells (7, 8). Initially, very promising as an asthma biomarker (8), periostin failed to become a surrogate for sputum eosinophilia (9), but it seems to be a good biomarker for airway remodeling (10, 11) and can differentiate between T2 and non-T2 asthmatics/COPD (12).

Another important question in clinical practice is the stability of asthma phenotypes over time. Although in the study of Woodruff et al. (6), phenotypic markers were reproducible and subjects who were T2 or non-T2 at baseline remained T2 and non-T2 on repeated evaluation at 8 weeks, no studies have been conducted to assess the stability of these phenotypes over longer periods of time. Therefore, to overcome all these doubts about the implied phenotypical approach, Wenzel proposed a new management of asthmatic phenotypes: evolution from the clinical approach to molecular mechanisms to finally reach the endotype (13). The term “endotype” is much more ambitious and exact than phenotype, as it is defined as a subtype of the asthmatic disease determined by a unique and differentiated functional or pathophysiological mechanism (14). Although endotypes might exceptionally shift or really overlap, coexisting in the same patient, as they are defined by a baseline mechanism, they are generally stable and produce more useful results. Precision medicine implies targeting the endotype, the mechanism.

In a clinical scenario of such heterogeneity and lack of other useful and available biomarkers for patients with uncontrolled moderate-to-severe asthma, the aims of this study were to (1) assess the stability over time of molecular T2 and non-T2 phenotypes, defined according to the simple biomarkers proposed by Woodruff (serum IgE levels and peripheral blood eosinophilia); (2) assess the correlation of these phenotypes with serum periostin levels; and (3) assess the correlation between serum periostin and asthma endotypes, on the path to personalized medicine in asthma.

## 2. Materials and methods

### 2.1. Study design and participants

We designed an ambispective study conducted at the Severe Asthma Unit of the Hospital Universitario 12 de Octubre (Madrid, Spain). Patients previously diagnosed with moderate-to-severe partially or uncontrolled asthma, according to current guidelines (15), and who attended follow-up visits were screened for T2 or non-T2 phenotypes based on retrospective biomarkers determinations; and then followed prospectively for a minimum of 1 year when biomarkers were reassessed and the stability of the phenotype was analyzed. Inclusion criteria were the following: a minimum of one complete blood cell count and a serum total IgE level for the assessment of the initial T2 asthma phenotype; written informed consent for participation in the study. As the study aimed to assess phenotype stability, as defined by blood biomarkers and their correlation with periostin, we excluded all current and future predictable conditions or treatments that might have interfered with biomarkers levels. Thus, exclusion criteria were the following: checked at both moments (at the initial evaluation and at least 1 year after the follow-up visit); patients with any concomitant malignancy (<5 years), pregnancy, infection, trauma, or surgery (30 days), patients treated with omalizumab or other biologics (6 months or 5 half-lives of the drug), systemic corticosteroids (30 days), specific allergy immunotherapy (5 years); and patients with asthma so uncontrolled at baseline that implied imminent starting on a biological treatment or entering a clinical trial. All patients who fulfilled all inclusion and exclusion criteria were consecutively included in the study.

The study was conducted in accordance with the Declaration of Helsinki (6th World Medical Assembly 2013) and was approved by the Clinical Research Ethics Committee of the Hospital Universitario 12 de Octubre (N°14/368).

### 2.2. Definitions and study procedures

Asthma: Diagnosed according to the Spanish Guidelines (15) for the presence of symptoms of wheeze, breathlessness, and cough plus a positive bronchodilator test (significant improvement by >12% and 200 mL in the forced expiratory volume in 1 s[FEV1] 10 min after the inhalation of 200 μg of salbutamol) or a positive methacholine test [a provocative concentration of methacholine required to lower the FEV1 by 20% (PC20) of <4 mg/mL].

Atopy: At least one positive skin prick test/specific IgE to common aeroallergens, independent of the presence or absence of clinical symptoms correlated to the respective sensitization (16).

Allergy: The presence of suggestive clinical symptoms supported by positive skin prick tests and/or positive specific serum IgEs to the suspected allergen (16).

### 2.3. T2/non-T2 asthma molecular phenotypes

Patients were assigned to the T2 or non-T2 phenotype group based on serum total IgE and peripheral eosinophil levels established by Woodruff et al. (6): the T2 phenotype was defined by the concurrent presence of total IgE ≥ 100 IU/mL and eosinophils ≥0.14 × 10^9^/L in peripheral blood. Patients with only one or none of the above criteria met were defined as having a non-T2 phenotype. The initial phenotype was established retrospectively according to data recorded in the patient's medical record. After inclusion in the study, patients were followed prospectively, and analyses of total IgE levels and blood eosinophil count were repeated after a minimum interval of 1 year from the inclusion visit.

### 2.4. Endotypes

Patients were also classified according to asthma endotypes in the groups of allergic asthma, aspirin-exacerbated respiratory disease (AERD), late-onset eosinophilic asthma, and non-eosinophilic asthma, according to thorough clinical history and the other criteria proposed by Lötvall et al. (14).

– Allergic asthma: suggestive clinical history, confirmed by concordant positive skin prick tests and/or specific IgEs to the suspected allergens. Allergic asthma, by definition, implies atopy and usually eosinophilia, but the latter is variable.
– AERD (aspirin-exacerbated respiratory disease): typically late onset, associates chronic rhinosinusitis with or without nasal polyps, aspirin intolerance confirmed by oral positive challenge with aspirin/other COX-1 inhibitors. It may associate atopy or coexist with a real allergic endotype in the same patient.
– Late-onset eosinophilic asthma: patients who meet eosinophilic asthma criteria (blood eosinophils >0.26 × 10^9^/L (17) are non-allergic non-AERD; typically late onset, associates chronic rhinosinusitis with or without nasal polyps and aspirin tolerance. Usually, it is non-atopic, although some patients might show subclinical sensitization to inhaled allergens.
– Non-T2, non-allergic, non-eosinophilic asthma: patients with no evidence of eosinophilic asthma (<140/mm^3^) or T2 inflammation, without suggestive clinical symptoms of allergic asthma (6).

Other data, including demographics, asthma characteristics, smoking history, lung function, and skin prick tests, were collected at baseline and follow-up visits.

Blood samples were obtained by venipuncture using a serum tube without an anticoagulant. Serum was collected, placed into aliquots, and immediately frozen at −80°C until analysis. Periostin levels were measured using a streptavidin–HRP ELISA commercial kit (DuoSet ELISA, R&D Systems, Minneapolis, MN, USA) according to the manufacturer's instructions. The specificity of the periostin assay is 100%, and the detection limit is 180 pg/mL. In all patients, serum periostin levels were measured in duplicate (11).

### 2.5. Statistical analysis

Categorical variables were expressed as frequencies and percentages, and quantitative variables were expressed as means and standard deviations (SDs). Quantitative variables were compared with Student's *t*-test or the Mann–Whitney *U*-test, according to the normal or not normal distribution of data. McNemar's test was used to compare the percentage of patients with T2 and non-T2 phenotypes at baseline and follow-up. The correlation between serum periostin levels and the study variables, including T2 phenotypes, asthma endotypes, gender, smoking status, age at the onset of asthma, pulmonary function tests, fractional exhaled nitric oxide (FeNO) atopy, serum IgE levels, and eosinophil blood count, was assessed with Pearson's correlation coefficient. Statistical significance was set at *P* < 0.05.

## 3. Results

A total of 53 patients, 38 women and 15 men, with a mean age of 47 years (range 16–77 years), were included in the study. The baseline characteristics of the patients are shown in Table 1. Thirteen patients (24.5%) were current smokers, and 34 (64.1%) showed at least one positive skin prick test. The forced expiratory volume in 1 s (FEV_1_)/postbronchodilator forced vital capacity (FVC) ratio was <70% in 44% of the patients. In relation to asthma endotypes, allergic asthma was diagnosed in 16 patients, AERD in 14, late-onset eosinophilic asthma in 4, and non-T2 non-eosinophilic asthma in 19 patients.

**Table 1:** Demographic data and characteristics of the 53 patients included in the study.

Variables	No. of patients (%)
Gender	
Male	15 (28.3)
Female	38 (71.7)
Smoking status	
Current smoker	13 (24.5)
Never smoker	31 (58.5)
Ex-smoker	9 (17.0)
Age at the onset of asthma	
<12 years old	9 (17.3)
>12 ≤40 years old	22 (42.3)
>40 years old	21 (40.4)
Atopy (at least one positive skin prick test to common aeroallergens)	
Present	34 (64.2)
Absent	19 (35.8)
T2 phenotype	
T2	21 (39.6)
Non-T2	32 (60.4)
Asthma endotype	
Allergic asthma	16 (30.2)
Aspirin-exacerbated respiratory disease	14 (26.4)
Late-onset eosinophilic asthma	4 (7.5)
Non-eosinophilic asthma	19 (35.8)

T2 phenotypes were stable: there were no significant differences in the percentage of patients with T2 and non-T2 phenotypes at the initial evaluation and the follow-up visit. At the initial assessment, the T2 phenotype was found in 41.5% (*n* = 22) patients and the non-T2 phenotype was found in 58.4% (*n* = 31). In the second evaluation, at least after 1 year of follow-up, the non-T2 phenotype of only 1 patient changed to a T2 phenotype, thus resulting in 23 patients (43.4%) in the T2 group and 30 (56.7%) in the non-T2 group (*P* = 0.012). Sixty percent of patients were followed for 3 years prospectively, while retrospective data were available and analyzed for 20% of the patients for 5 years, and for the other 20%, data were available for a variable period between 6 and 20 years. During all these periods, the phenotypes remained stable. One patient was initially misclassified as non-T2 asthma at the beginning of the study; in the follow-up visit, he was correctly diagnosed with occupational asthma due to isocyanates plus probable asthma-COPD overlap.

**Figure 1:**
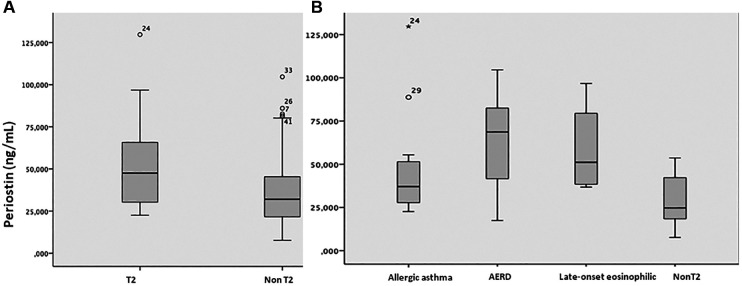
Serum periostin levels in the different asthma (**A**) phenotypes and (**B**) endotypes. T2, T2 asthma phenotype; Non-T2, non-T2 asthma phenotype; AERD, aspirin-exacerbated respiratory disease.

Serum periostin levels were higher among patients with the T2 phenotype [mean (SD): 52.7 (26.2) ng/mL] than those with the non-T2 phenotype [mean (SD): 52.7 (26.2) vs. 39.1 (25.6) ng/mL], but the difference was not statistically significant (*P* = 0.063) (see Figure 1A, Table 2). Nevertheless, periostin levels were significantly different (*P* = 0.001) according to endotypes, with mean values of 45.7 (27.9) ng/mL in allergic asthma, 64.7 (24.9) ng/mL in AERD, 59.0 (27.6) ng/mL in late-onset eosinophilic asthma, and 28.3 (13.3) ng/mL in non-eosinophilic asthma (see Figure 1B, Table 2).

**Table 2:** Serum periostin levels according to asthma phenotypes and endotypes.

Pheno/endotypes	Serum periostin, mean (SD) [range: minimum–maximum], ng/mL	*P-*value
Phenotype		
T2 (*n* = 22/23^a^)	52.7 (26.2) [22.6–129.7]	0.063
Non-T2 (*n* = 31/30^a^)	39.1 (25.6) [7.7–104.6]	
Endotype		
Allergic asthma (*n* = 16/17^a^)	45.7 (27.9) [22.6–129.7]	0.001^b^
Aspirin-exacerbated respiratory disease (*n* = 14)	64.7 (24.9) [17.4–104.6]	
Late-onset eosinophilic asthma (*n* = 4)	59.0 (27.6) [36.8–96.8]	
Non-eosinophilic asthma (*n* = 19/18^a^)	28.3 (13.3) [7.7–53.6]	

^a^ Number of patients in the second IgE and blood eosinophils measurement

^b^ Statistically significant difference.

**Figure 2:**
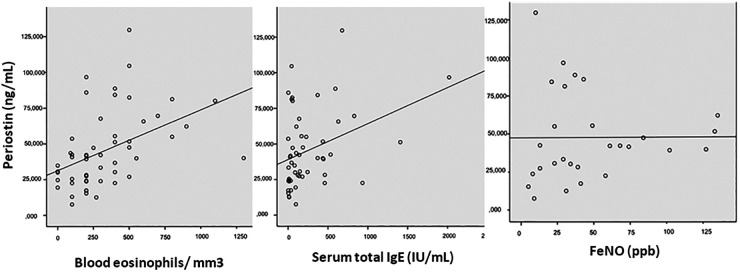
Correlation between serum periostin levels and FeNO, total serum IgE, and blood eosinophilia. FeNO, exhaled fraction of nitric oxide.

No correlation was observed between serum periostin and gender, smoking habit, or the age at the onset of asthma. Serum periostin levels were slightly higher (ns) in non-atopic patients [54.5 (27.6) ng/mL, range 7.7–104.6 ng/mL] than in those with at least one positive skin prick test [40.5 (25.0) ng/mL, range 7.7–129.7 ng/mL] (*P* = 0.06). No correlation was found between serum periostin and the other classical T2 biomarkers—total IgE levels, blood eosinophil count, and FeNO (see Figure 2).

## 4. Discussion

Previous studies on the stability of asthma phenotypes have shown inconsistent results (18–20). Kupczyk et al. (21) assessed the stability of phenotypes defined by either biomarkers or other variables in 169 asthma patients of the Pan-European BIOAIR cohort. After 1 year of follow-up, the allocation to clusters was changed in 23.6% of asthma patients when defined by phenotypes and, remarkably, in 42.3% of patients when stratified according to sputum inflammatory cells. In the elegant study of Woodruff et al. (6), the stability of T2 and non-T2 profiles based on epithelial gene expression was demonstrated, even in the groups of patients treated with inhaled fluticasone or matched placebo. In our study, in which the stability of asthma phenotypes has been assessed in a simplified manner using total serum IgE and eosinophil count, the percentage of patients assigned to the T2 and non-T2 groups remained practically unchanged after a follow-up of 1 year. In contrast to the systemic use of corticosteroids, treatment with inhaled steroids was not an exclusion criterion of the study. The main finding of our study, the stability of asthma phenotypes over 1 year, is clinically relevant, especially because the standard laboratory tests for the measurement of total IgE and blood cell count are easily available in daily practice. The long follow-up period is another strength of our study. Although a minimum 1-year follow-up was established between the initial and the second blood sampling, the extent of total follow-up was longer than 3 years in all patients and between 6 and 20 years in 20% of them. However, the present findings should be interpreted taking into account the small study population and the ambispective design.

In our study, serum periostin levels were not associated with the T2 or non-T2 phenotypes, although a tendency to higher levels was observed among patients with the T2 phenotype. The statistical significance, one might argue, has not been reached because of the insufficient sample size: In larger studies (*N* = 292), in which our group participated, periostin did differentiate between T2 and non-T2 asthmatics/COPD (12). Nonetheless, both in the current study and the CHACOS study, there is a significant overlap in mean periostin values between the molecular T2 and non-T2 phenotypes. In the CHACOS study, the mean periostin level was 39.73 ng/mL (28.05–54.40) in the T2 phenotype vs. 34.20 (26.94–41.43) in the non-T2 phenotype, *P* = 0.005. With an area under the ROC curve (AUC) of 0.602 (IC: 95 0.528–0.677), periostin did not yield enough sensitivity and specificity to accurately predict T2 and non-T2 molecular phenotypes, with the best cutoff of 35.2 ng/mL (12). Therefore, the sample size does not seem to be the problem but the actual low sensitivity of the test.

On the contrary, significant differences in serum periostin levels were found according to asthma endotypes, with the highest levels in patients with AERD, which is consistent with data obtained in other studies (22). Kim et al. (22) measured serum periostin levels in 227 adults with asthma and compared the results between patients with AERD and aspirin-tolerant asthma (ATA) and between patients with eosinophilic and non-eosinophilic asthma. In their study, serum periostin levels were significantly higher in patients with AERD vs. ATA, in patients with severe asthma vs. non-severe, and in patients with eosinophilic vs. non-eosinophilic asthma. It is known that there is a more intense eosinophilic infiltration in the lower and upper airway mucosa and upregulation of eosinophil-related cytokines in AERD (23, 24), which is closely associated with comorbid conditions such as chronic rhinosinusitis and/or nasal polyps and more severe asthma. In this respect, and emphasizing the importance of determining the endotype with the aims of precise and even personalized medicine, we consider it would be useful to determine serum periostin levels in individual patients at the time of assessing a biological therapy (anti-IgE, anti-IL-5, and anti-IL-4/13). In an individual patient with AERD, for instance, who is most surely eosinophilic, a low periostin level implies probably choosing an anti-IL-5, while a high periostin might predict better response to an anti-IL-4/13 (8, 25–27).

On the other hand, of the 34 atopic patients (defined as sensitized to at least one aeroallergen in the skin prick tests), only half of them showed an allergic asthma endotype, and 32.3% (11 out of 34 patients) were diagnosed with non-eosinophilic asthma. Also, a patient with negative skin prick tests to common aeroallergens was diagnosed with allergic occupational asthma to isocyanates (positive specific IgEs in serum). Results of diagnostic tests should always be analyzed and reported with propriety since atopy is not a definitive indicator of allergic asthma and apparently not even of eosinophilic asthma. The results of our study emphasize the heterogeneity within the T2 phenotype and the need for more personalized approaches in asthma. One of the strengths of our study resides precisely in the clear definitions of atopy, allergy, allergic and non-allergic asthma, eosinophilic asthma, and T2 asthma, which are not synonymous terms. Although these traits share some common underlying mechanisms, the genetic and epigenetic interindividual variety, in terms of T2 genes, is probably what makes asthma patients so heterogeneous; therefore, we think these traits and their clinical significance should be analyzed one by one in each patient. Yet, in multiple asthma studies or clinical trials with new drugs, these terms are sometimes inaccurately exchanged and analyzed, inducing more confusion in the already complex decision-making process of choosing the ideal biologic for a real-life patient.

In sum, this study shows that T2 and non-T2 asthma phenotypes assessed by accessible methods in daily practice are stable over time. Serum periostin levels were slightly higher (ns) in patients with the T2 phenotype but strongly correlated to asthma endotypes, which may contribute to guide therapies targeting T2 cytokines in a more individualized fashion. Periostin is not an ideal biomarker, but it maintains a certain value when interpreted properly, together with the classical T2 biomarkers. It seems that, as with other biomarkers such as FeNO, clinicians need to learn how to interpret periostin values in the context of the patient's characteristics and comorbid diseases (28). Also, measuring serum periostin with a common commercial ELISA kit is neither complicated nor expensive; hence, it could be performed routinely in selected patients in severe asthma units.

From a practical point of view, based on the present study, we propose a simple two-step algorithm for approaching biological therapy of severe asthma: step 1—classifying in T2 and non-T2 molecular phenotypes; step 2—endotyping as described, and within the same endotype, individual characterization and interpretation of available biomarkers and their dynamics, including periostin. Further validation is, of course, required to examine the effectiveness of this algorithm, but we consider it would be a practical step forward on the path to personalized medicine in asthma.

## Data Availability

The raw data supporting the conclusions of this article will be made available by the authors without undue reservation.
